# Perioperative Management of a Patient with Secreting Paraganglioma Undergoing Cesarean Section

**DOI:** 10.1155/2022/9065324

**Published:** 2022-03-07

**Authors:** Ana Rita Bettencourt, Catarina Alves

**Affiliations:** Anesthesiology Resident, Anesthesiology Department, Hospital Garcia de Orta, Almada, Portugal

## Abstract

Paraganglioma is a catecholamine-secreting tumor (CST) in extra-adrenal autonomic ganglia and a rare cause of hypertension during pregnancy. If not properly treated, it can lead to disastrous outcomes for both the mother and fetus. This report describes the successful anesthetic management of a paraganglioma diagnosed during pregnancy. A pregnant woman, with 32 weeks of gestational age, presented with severe paroxysmal hypertension, refractory to methyldopa and nifedipine at maximum dosages, headache, sweating, and palpitations. Diagnostic work-up was positive for elevated serum and urinary normetanephrines, and magnetic resonance showed a solid nodule above the hilum of the right kidney, suggestive of paraganglioma. Optimal alpha-blockade was achieved with doxazosin, and given the advanced gestational age, tumor resection was postponed until after delivery. Cesarean delivery was scheduled at 34 weeks, under combined spinal-epidural anesthesia and continuous blood pressure monitoring. Antihypertensive drugs were prepared for immediate administration as needed. Intraoperative and postoperative periods went uneventfully for both the mother and newborn, both under intensive care observation for 24 h.

## 1. Introduction

While hypertension occurring during pregnancy is generally associated with a hypertensive disorder of pregnancy, less-frequent secondary etiologies should be screened according to hypertension severity and accompanying symptoms [1]. One of the most important diagnostics to rule out are catecholamine-secreting tumors (CSTs), such as pheochromocytoma or paraganglioma, frequently presenting with paroxysmal symptoms such as headaches, sweating, and palpitations. If not timely diagnosed and treated, maternal and fetal mortality can reach up to 40 to 50% [2–8]. Symptomatic CST in pregnant women are rare [2], with its incidence from 1 in 15,000 to 54,000 pregnancies [3].

Even though the rule is to proceed to tumor resection shortly after optimal alpha-blockade, when the diagnosis occurs during pregnancy, there are not clear guidelines as to the timing of tumor resection versus timing of delivery, type of delivery, anesthetic management, and postoperative care [9].

This case report describes the successful anesthetic management of a patient diagnosed with paraganglioma during pregnancy.

## 2. Case Presentation

A 34-year-old woman, at 32 weeks of gestational age, with a history of a previous uneventful pregnancy, presents with new onset severe paroxysmal hypertension associated with headache, sweating, palpitations, and light-headedness. There was no relevant family history of high blood pressure, genetic syndromes, or endocrine tumors.

Even though she was started on methyldopa 500 mg three times a day and nifedipine 30 mg two times a day, the patient maintained frequent, symptomatic hypertensive paroxysms. For this reason, she was admitted for continuous monitoring, blood pressure control, and diagnosis in a tertiary hospital. Monitoring included continuous cardiotocography, obstetric ultrasound, maternal electrocardiography, and regular noninvasive blood pressure monitoring. During this time, the patient suffered hypertensive paroxysms (185/117 mmHg, heart rate maximum of 160 bpm), facial flushing, and severe headache, with no signs of fetal distress. Screening for secondary hypertension revealed increased serum and urinary normetanephrines (909 pg/mL, normal values inferior to 111 *μ*g/mL and 5179 *μ*g/24 h, normal range between 162 and 527 *μ*g/24 h) and serum and urinary metanephrines in the normal range (33 pg/mL, normal values inferior to 64 *μ*g/mL and 72 *μ*g/24 h, normal range between 329 and 1263 *μ*g/24 h). Other biochemical data were in the normal range, including fasting glucose level of 78 mg/dL, potassium of 4.2 mEq/L, and urea of 34 mg/dL. Magnetic resonance showed a solid nodule with T2 hypersignal and heterogenous signal T1 above the hilum of the right kidney, 41 × 53 × 53 mm in size, suggestive of paraganglioma.

Following this diagnosis, the patient was started on doxazosin, titrated to optimal alpha-blockade (final dose 8 mg/day), achieving normotension and resolution of associated symptoms.

Given that the patient was in her third trimester and considering both the risk of inducing premature labor with tumor resection as well as the repercussions of hemodynamic instability for both the mother and fetus, it was decided to have the cesarean scheduled at 34 weeks of gestation and tumoral resection 6 weeks afterwards. Doxazosin was maintained throughout the perioperative period, and a postoperative Intensive Care Unit (ICU) bed was provided. Before induction, an arterial line was placed and antihypertensive intravenous drugs, such as labetalol and dinitrate isosorbide, were prepared to rapidly control intraoperative hypertension.

When positioning in lateral decubitus, the patient became hypertensive, with a systolic blood pressure of 200 mmHg and heart rate of 80 bpm, controlled with labetalol bolus total of 20 mg. A combined spinal-epidural was performed, and 8 mg of bupivacaine and 3* μ*g of sufentanyl were administered intrathecally. The remaining intraoperative period was uneventful. The newborn had an Apgar score of 9/10, weighed 2286 grams, and was transferred to a neonatal intensive care unit for monitoring for 24 h. There was no need for respiratory/circulatory support or other complications regarding both the mother and fetus. The tumor resection was performed as planned at 6 weeks postpartum, under general anesthesia. Anatomopathology of the tumor confirmed the diagnosis of paraganglioma, with low proliferative indexes, vascular and capsular invasion, and safe surgical margins. Postoperative metanephrine and normetanephrine levels were in the normal range, and genetic study was positive for germinal mutation of SDHB gene.

## 3. Discussion

This report highlights the optimal perioperative management and safety of neuraxial blockade as a key to safely carry out cesarean section in a patient with CST. This involves a multidisciplinary team of obstetrician, anesthesiologist, general surgeon, endocrinologist, neonatologist, and intensivist.

CST can manifest during pregnancy and have a high maternofetal morbimortality rate, if not timely diagnosed or adequately treated [2–8]. Maternal risks of CST include major adverse cardiovascular events and death [6]. Sustained high levels of catecholamines affect the fetus indirectly through maternal distress and directly from intense vasoconstriction of uteroplacental circulation, causing intrauterine growth restriction and fetal hypoxia [3]. Alpha-blockade should be started as soon as possible with either phenoxybenzamine or a competitive selective *α*-1 antagonist, such as doxazosin. Disadvantages of the use of phenoxybenzamine include potential hemodynamic stress and respiratory depression to the newborn, as it crosses the placenta barrier [10], and prolonged hypotension after tumoral resection, due to its long-lasting noncompetitive nonselective *α* blockade. Doxazosin also crosses the placenta but appears to be safer to the newborn [11]. Following alpha-blockade, beta-blockers may also be initiated in order to manage tachycardia. In the intraoperative setting, severe hypertension may result from anxiety, nociceptive stimuli, and tumoral manipulation. This requires the anesthesiologist to have short-acting antihypertensive agents prepared for administration, such as beta-blockers (labetalol and esmolol) or vasodilators (dinitrate isosorbide). This will rapidly control high blood pressure and lessen the severity of hypotension after tumor resection.

Gestational age at the time of diagnosis and symptom control affect the decision on timing of surgery. Several strategies have been described in the literature, namely, whether to promptly perform tumor excision, wait and combine with a cesarean section, or even to delay it until after birth [9, 12, 13]. If diagnosis and optimal medical treatment are achieved in the first trimester, the most recommended option is to proceed to surgery, considering the low risk of inducing premature labor. After 30 weeks of gestation, the risk of inducing labor by having elective surgery is significant, and so is the risk of poor maternofetal outcomes. In this case, the decision was made to delay tumoral resection and proceed to delivery by cesarean at 34 weeks.

In summary, CSTs during pregnancy are a rare condition, with recognized potential for adverse perioperative events. Early diagnosis and careful perioperative management within a multidisciplinary team is essential to have the best possible outcomes. Anesthesia for patients with CST requires eviction of sympathetic stimuli, invasive blood pressure monitoring, hemodynamic monitoring in the postoperative period, and good-quality analgesia.
